# A Ship Trajectory Prediction Framework Based on a Recurrent Neural Network

**DOI:** 10.3390/s20185133

**Published:** 2020-09-09

**Authors:** Yongfeng Suo, Wenke Chen, Christophe Claramunt, Shenhua Yang

**Affiliations:** 1 Navigation College, Jimei University, Xiamen 361021, China; 201811823002@jmu.edu.cn (W.C.); shyang@jmu.edu.cn (S.Y.); 2 Naval Academy Research Institute, BP 600, 29240 Brest Naval, France; christophe.claramunt@ecole-navale.fr

**Keywords:** trajectory prediction, deep learning, DBSCAN, GRU, LSTM, redundant data

## Abstract

Ship trajectory prediction is a key requisite for maritime navigation early warning and safety, but accuracy and computation efficiency are major issues still to be resolved. The research presented in this paper introduces a deep learning framework and a Gate Recurrent Unit (GRU) model to predict vessel trajectories. First, series of trajectories are extracted from Automatic Identification System (AIS) ship data (i.e., longitude, latitude, speed, and course). Secondly, main trajectories are derived by applying the Density-Based Spatial Clustering of Applications with Noise (DBSCAN) algorithm. Next, a trajectory information correction algorithm is applied based on a symmetric segmented-path distance to eliminate the influence of a large number of redundant data and to optimize incoming trajectories. A recurrent neural network is applied to predict real-time ship trajectories and is successively trained. Ground truth data from AIS raw data in the port of Zhangzhou, China were used to train and verify the validity of the proposed model. Further comparison was made with the Long Short-Term Memory (LSTM) network. The experiments showed that the ship’s trajectory prediction method can improve computational time efficiency even though the prediction accuracy is similar to that of LSTM.

## 1. Introduction

AIS data provide real-time trajectory data which can be used to monitor ship’s navigation status and trigger alert mechanisms for ship collision avoidance, maritime monitoring, trajectory clustering, ship traffic flow predicting, and maritime accident investigations [1]. The most basic information from AIS data gives spatiotemporal data composed of time and location, which is usually plotted on an electronic chart that can then form a ship trajectory.

In sea areas or ports with high traffic density and complicated conditions, a key issue is to improve the safety of ships sailing at sea. Vessel Traffic Service (VTS), whose objective is to accurately and effectively monitor and predict ship trajectories and real-time ship trajectories, provides a valuable technical support for early warning of marine traffic accidents [2]. To improve the safety of ships sailing in the environment of complex and changeable seas, it is necessary to provide trajectory prediction and danger warning functions to a ship’s intelligent navigation system. However, the maritime navigation environment is prone to many incidents, especially in crowded port waters, and it is not easy to predict moving targets.

In a related work, a Bayesian probability trajectory prediction model based on a Gaussian process is introduced. A K-order multivariate Markov chain is applied to establish a state transition matrix to train a large amount of data and support short-term prediction of ship positions, but this approach is still sensitive to previous ship positions this resulting in low accuracy [3]. A single ship neighborhood approach based on historical data has also been suggested. The search algorithm can support short-term prediction, but it cannot deal with the branch trajectory problem [4].

The research presented in this paper introduces a deep learning theoretical framework and a Gate Recurrent Unit (GRU) model to predict vessel trajectories. The approach is based on an integration, clustering, and correction of maritime navigation trajectories. A recurrent neural network is applied to predict and train real-time ship trajectories. Ground truth data from AIS raw data in the port of Zhangzhou, China were used to train and verify the validity of the proposed model. Further comparison was made with the Long Short-Term Memory (LSTM) network that has been widely used in the field of deep learning. The rest of the paper is organized as follows. Section 2 briefly introduces related work and the motivation of our work. Section 3 develops the main principles behind our data preparation approach. Section 4 presents the GRU model. Section 5 reports on the experiments. Section 6 concludes the paper and outlines further work.

## 2. Related Work

Trajectory prediction methods based on statistical methods are commonly used in the ship field, including Gaussian process regression models, which are the most common. Anderson [5] took time as the independent variable, obtained the measured value in discrete time, and regarded the trajectory as a one-dimensional Gaussian process. The method defines the prior continuous time through the nonlinear time-varying stochastic differential equation driven by white noise, calculates the posterior distribution of the predicted value by obtaining the joint prior density and covariance matrix of the observed value and the predicted value, and uses dynamics in combination, odel smooth trajectory estimation. Rong [6] decomposed ship motion into horizontal and vertical. In the lateral direction, a Gaussian process is used to model the uncertainty of lateral motion, and the longitudinal direction is estimated by acceleration. The horizontal distribution model obtains the hyperparameters of the Gaussian regression model through historical AIS data, and the mean function and covariance function are determined by the hyperparameters. The predicted trajectory can be estimated by evaluating the mean value and covariance matrix, that is, the mean value and variance are used to describe the ship’s lateral position and its uncertainty. The advantage of Gaussian process regression is its strong applicability and easy to understand. The disadvantage is that the amount of calculation is large, and as the forecasting time passes, the accuracy of the forecast results will be greatly reduced. Jiang [7] used a polynomial Kalman filter to fit the piecewise polynomial features of the ship’s track. Kalman Filter (KF) can estimate the state of the system under the condition of uncertain factors. It combines joint distributions at different times to estimate unknown variables. This method first uses polynomial fitting to obtain the state equation and observation equation; determines the initial state, error covariance matrix, and other parameters; and then it completes the first step of filtering. Next, it performs N-step prediction and compares the predicted value with the true value. The algorithm iterates N times to update the error covariance matrix of the track points to complete the trajectory prediction.

The Kalman filter implements this algorithm in the form of recursion. Its advantage is that it occupies less storage space during the calculation process and can realize short-term trajectory prediction. The disadvantage is that the initial state of the model and the assumptions under ideal conditions have a significant impact on the accuracy of prediction.

Neural networks are computing systems with interconnected nodes that work similar to neurons in the human brain. Using algorithms, they can recognize hidden patterns and correlations in raw data, cluster and classify them, and—over time—continuously learn and improve [8]. Neural networks are also ideally suited to solving complex problems in real-life situations. They can learn and model the relationships between inputs and outputs that are nonlinear and complex; make generalizations and inferences; reveal hidden relationships, patterns, and predictions; and model highly volatile data (such as time series data) [9]. With the popularization of artificial intelligence, neural networks have also been gradually applied to the field of maritime navigation [10,11]. Historical ship trajectory data and trajectory characteristics are used as an input, while predicted ship trajectory data are the output of the neural network [11]. Zhang et al. proposed a deep learning method that integrates multiple ship movements, which can be adapted to predict different many categories of ship trajectories after training the neural network appropriately [12]. Overall, the resulting accuracy varies as a function of ship categories, thus the modeling approach still has to be improved.

Brian [13] proposed a dual linear autoencoder method to predict the future trajectory of ships. Autoencoder (AE) includes two processes of encoding and decoding, which can extract hidden features in the data and reconstruct the original input data with new features. The autoencoder can send the extracted new features to the supervised learning model and then use them to predict the trajectory. This model first clusters ship trajectories based on historical AIS data to predict the trajectories of ships in the selected category. This method generates the entire ship trajectory, rather than the iterative state based on historical predictions. Through the potential distribution of possible future trajectories of ships, the model can predict multiple ship trajectories and predict their uncertainties. The autoencoder can extract deep-level data features. In addition, using sparse autoencoder can obtain better initial parameters in engineering applications. However, the extraction of deep-level data features requires accurate grasp. Excessive extraction will lead to the extraction of useless data features, making the model effect poor.

Mao [14] proposed an ELM-based marine trajectory prediction method to predict the trajectory of ships. Extreme Learning Machine (ELM) is a machine learning algorithm based on a single hidden layer feedforward neural network. Since the ELM algorithm does not require the weights and biases of the iterative neural network, the training time of the ELM neural network is less than that of the traditional neural network. The advantage of this algorithm is that the generalization performance of the model is better, and the calculation speed can be improved. However, it is more dependent on the number of model nodes and the selection of some optimal parameters.

A “sequence-to-sequence” recurrent neural network model has been developed to mesh and serialize a ship trajectory into a neural network model, in order to predict the main trajectory and arrival time [15]. A LSTM model has been introduced to predict ship’s position by evaluating the probability distribution and obtains relatively valid results [16]. To improve the accuracy of the prediction mechanisms, a multiple azimuth autonomous device sensor has been used as an additional data input, but the approach relies on a large amount of AIS data so computational performance is relatively low [17]. While large AIS historical data can be used as references for predicting maritime trajectories, data quality is often not guaranteed, and data redundancy is a major problem: worldwide there are about 1600 AIS receivers on the coastline of more than 150 countries and 65,000 ships sailing around the world [18]. Furthermore, abnormal data due to either environmental conditions or technical issues are likely to generate significant trajectory prediction errors [19,20]. The most appropriate balance between the necessary training of the prediction mechanisms with large enough trajectory data, and the negative impact of redundant and noisy data is still a crucial issue for practicality [21]. The advantages of common statistics-based methods are that the data occupy less storage space during the calculation process, can realize short-term trajectory prediction, and the calculation method is relatively lightweight, achieving an effect similar to deep learning. The disadvantage is that the initial state of the model and the assumptions under ideal conditions have a large impact on the prediction results. However, statistical methods cannot learn the effects of shallow reefs, islands, and other spatial factors on the trajectory of ships as deep learning. This feature makes deep learning more practical in trajectory prediction. This motivates our search for a trajectory prediction approach that will take into account the impact of redundant and noisy data on the neural network training and optimize the input trajectory dataset to improve the final quality of the modeling approach. This leads us to combine a neural network with a GRU framework and that is introduced in the following sections. 

## 3. Modeling Approach

**Figure 1:**
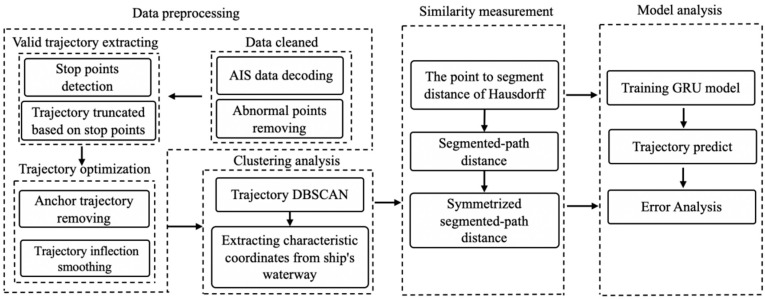
Flowchart of the vessel trajectory prediction framework.

The prediction model framework consists of four parts: data preprocessing, clustering analysis, similarity measurement, and model analysis (Figure 1). Data preprocessing is an essential part of deep learning, as cleaned data can improve the model performance. Clustering analysis uses historical data to extract the main ship trajectory areas. Similarity evaluation is based on the symmetric segmented-path distance to eliminate the influence of a large number of redundant data. The model analysis applies the deep learning model of a lightweight recurrent neural network GRU to train the model and predict ship trajectories.

### 3.1. Data Preprocessing

The AIS is an essential component of modern vessel navigation systems, which are installed and widely used on vessels to enhance the ability to identify targets and mark the location. Raw AIS data are used to build a reference dataset and define a matrix of historic AIS data as follows:

(1) $$X_{T_{j}}={\left[X_{1}\;,\;X_{2},\;\cdots ,\;X_{N}\right]}^{T}$$

where N is the total number of AIS messages and

(2) $$X_{i}=\left[MMSI_{i}\;,\;t_{i}\;,\;p_{i}{}^{T},\;c_{i}\;,v_{i}\right]$$

i ∈ {1, 2,…,N} is a vector where $MMSI_{i},\;t_{i},\;p_{i}{}^{T}$, and $v_{i}$ represent, respectively, the Maritime Mobile Service Identity (MMSI), timestamp, location (WGS84 longitude and latitude), course over ground (COG), and speed over ground (SOG). The MMSI is a unique identifier for each vessel.

**Figure 2:**
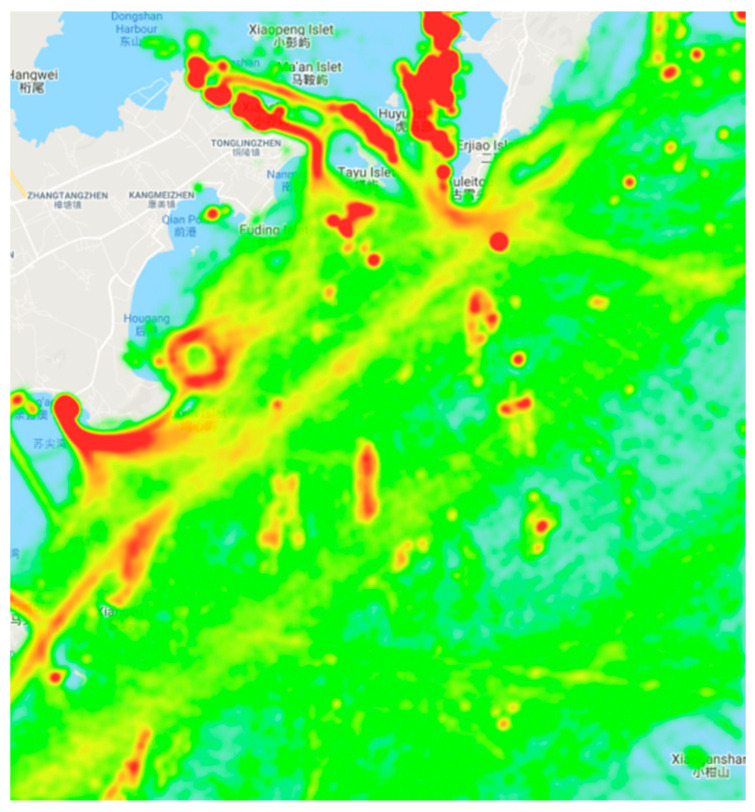
A visualization of the original AIS data in Zhangzhou, Fujian, China. The green line indicates the trajectory, and darker color indicates greater trajectory density. For example, the red area indicates the area with a large trajectory density.

**Figure 3:**
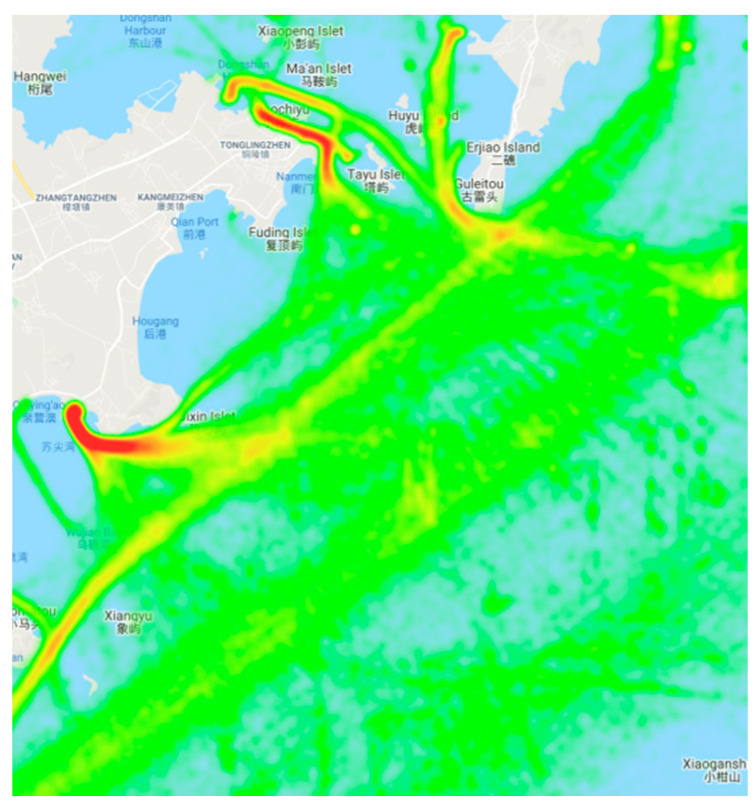
This is the heat map of the trajectory data processed by the algorithm; the trajectory in the state of drifting, anchoring, etc. is cleared, and the trajectory data in the normal sailing state is saved.

During the data preprocessing process, the wandering or anchoring trajectories in the original dataset are eliminated. We set the minimum time interval of the trajectory to 1200 s, because of the AIS information receiving interval is generally specified to be about 5–10 min, and the information interval higher than 20 min is used as the next stage of navigation status [16]. Each trajectory is optimized by a function Optimization ($V_{i}$) as shown in Algorithm 1. Figure 2 and Figure 3 show a comparison of two trajectories’ density heat maps before and after preprocessing. The green dots in Figure 2 show the location of the vessel, the red region indicates dense areas, and many error data or anchor trajectories are still contained. Figure 3 shows that green dots after processing are smooth and in sailing state, and yellow to red dots indicate denser trajectories. Many noisy data in the original data are eliminated. In the original ship data, the dense red ring area shown in Figure 2 contains data about many ships floating and anchoring. The speed of these ships is affected by wind and ocean currents and is often less than 1 knot, which is an abnormal navigation state. The data required for the experiment are the data of the ship in a sailing state; accordingly, a route performs smoothly along the motion trajectory, which makes each trajectory easier to analyze.
**Algorithm 1**: Data preprocessingInput: Original dataset $X_{T_{j}}$, minimum voyage time interval threshold ε. Output: Trajectories $T_{s_{i}}.$
1: Connect to the database.2: Get $V_{i}$ where i in Len(O) and $V_{i}$.//Δt is the total time interval of voyage i.3: if $R_{i}.mmsi$ = $R_{i-1}.mmsi\;\&\&R_{i}.sog<1kn\;\&\&\;\delta_{t}<1200s$ where $\delta_{t}$ = $R_{i}.t-R_{i-1}.t$//$R_{i}$ is the next state of $V_{i}$,$\;R_{i}.sog$ is the speed of the vessel, $\delta_{t}$ is the time interval between two-state.4: then Optimization ($V_{i}$), $T_{s_{i}}\to V_{i}$.//Optimize (·) is the trajectory optimizing function. Return:$\;T_{s_{i}}$


In Table 1, the total number of coordinates after preprocessing is reduced by nearly 71.8% compared to the initial data, and the number of trajectories is reduced by almost 92.1%, as only the vessels underway are saved as valid trajectories (e.g., anchor or wander is filtered). Nearly all noisy data have been eliminated from raw data, and more valid and regular vessel trajectories are obtained, which can improve the processing efficiency. 

**Figure 4:**
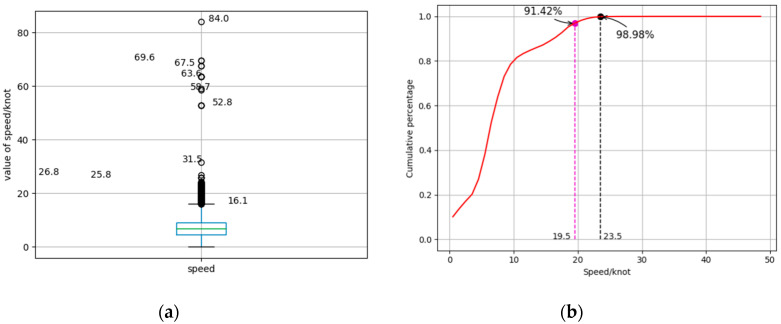
The two forms of AIS data ship speed’s distribution picture: (**a**) box diagram of ship speed, from which it can be seen that the speed is mainly distributed around 15, and a speed greater than 60 is an abnormal speed; and (**b**) probability distribution diagram, where normal data accounts for 98.98% of the total data.

During the actual reception and sending of AIS information in navigation state, due to the phenomenon of signal drift and artificial tampering of the AIS equipment, the received ship information is likely to show some abnormal locations, as illustrated in Figure 4. As shown in Figure 4a, the speed of the ship in the dataset is roughly concentrated at about 20 knot but also includes speed below 1 knot. In Figure 4b, the dataset contains some abnormal speeds greater than 20 knot. To restore the true state of the navigation, AIS information should be optimized to a valid trajectory which is approximated by

(3) $$P_{T_{j}}=\left[lat_{T_{j}},lon_{T_{j}},V_{T_{j}},\theta_{T_{j}},\omega_{T_{j}}\right]$$

while the average speed of the ship during a period can be estimated by

(4) $${\tilde{V}}_{T_{j}}=\frac{\sqrt{{\left(lat_{T_{j}}-lat_{T_{j+1}}\right)}^{2}+{\left(lon_{T_{j}}-lon_{T_{j+1}}\right)}^{2}}}{T_{j+1}-T_{j}}$$

where $\;lat_{m,T_{j}}$ and $lon_{m,T_{j}}$ are the longitude and latitude coordinates of the ship m at time $T_{j}$. At this time, the shipping speed should meet the condition given by

(5) $$V_{T_{j}}-a^{\prime }\left(T_{j+1}-T_{j}\right)\leq V_{T_{j}}\leq V_{T_{j}}-a\left(T_{j+1}-T_{j}\right)$$

where a and $a^{\prime }$ are the forward and reverse acceleration when the ship is moving forward. When the current data are identified as an abnormal speed, the average speed of the two points adjacent to the point is substituted to the data.

(6) $$V_{T_{j}}=\{\begin{array}{c} V_{T_{j}}\;,\;\;\;\;\;\;\;\;\;\;\;normal\;speed\;\;\\ {\tilde{V}}_{T_{j}}\;,\;\;\;\;\;\;\;\;\;abnormal\;speed \end{array}$$

When judging whether the data are tested as an inflection point or a sudden change point, we can approximate the location by

(7) $$\{\begin{array}{c} \hat{l}at_{T_{j}}=lat_{T_{j}}+V_{{\hat{T}}_{j}}\left(T_{j+1}-T_{j}\right)\\ \hat{l}on_{T_{j}}=lon_{T_{j}}+V_{{\hat{T}}_{j}}\left(T_{j+1}-T_{j}\right) \end{array}$$

where $lat_{m,T_{j}}$ and $lon_{m,T_{j}}$ are the position of the ship at time $T_{j}$. $\hat{l}at_{m,T_{j}}$ and $\hat{l}on_{m,T_{j}}$ give the location of the ship at time $T_{j+1}$. If the current location of the ship does not satisfy Equation (8), the current position information is replaced as follows:

(8) $$\sqrt{{\left(lat_{T_{j}}-lat_{T_{j+1}}\right)}^{2}+{\left(lon_{T_{j}}-lon_{T_{j+1}}\right)}^{2}}\leq 0.5a{\left(T_{j+1}-T_{j}\right)}^{2}$$

**Figure 5:**
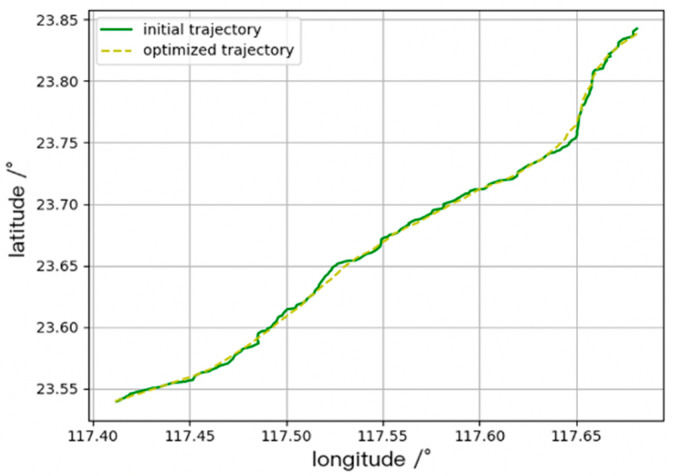
Plot of the comparison of the original trajectory and optimized trajectory of a same vessel.

Due to the non-equal interval of AIS data reception, when the data update interval is too low, the ship’s speed and acceleration are used to interpolate the course of the route following [9]. Figure 5 shows a comparison of two trajectories before and after optimization.

(9) $$lat_{T_{j}}=lat_{T_{j-1}}+\frac{1}{2}\cdot \Delta t\cdot \left[V_{T_{j}}+\Delta t\cdot a_{T_{j-1}}\cdot \cos\left(\frac{\pi \theta }{180}+\Delta t\cdot \omega_{T_{j-1}}\right)\right]$$

(10) $$lon_{T_{j}}=lon_{T_{j-1}}+\frac{1}{2}\cdot \Delta t\cdot \left[V_{T_{j}}+\Delta t\cdot a_{T_{j-1}}\cdot \sin\left(\frac{\pi \theta }{180}+\Delta t\cdot \omega_{T_{j-1}}\right)\right]$$

### 3.2. Cluster Analysis

This section introduces the trajectory clustering model based on the AIS data that were previously processed to generate regular shipping route patterns. 

The DBSCAN algorithm is a density-based point or line clustering algorithm that was introduced by Ester et al. [22]. The main advantage of this algorithm is that arbitrarily shaped clusters can be identified. By applying the DBSCAN algorithm, lines are mainly divided into three types: density-connected lines, outliers, and core lines. A line is generally classified as a core line if a minimum number of MinPts lines are included within a distance Eps, and the lines that are density connected with others are regarded as the same cluster, while points which are not density connected are regarded as outliers [23].

**Figure 6:**
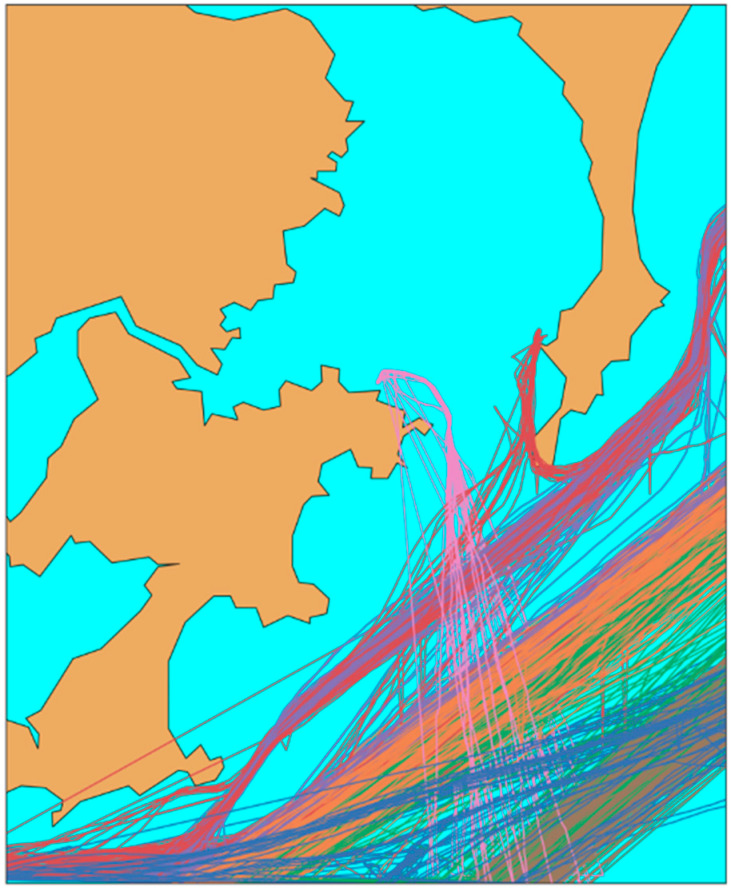
Trajectory clusters by DBSCAN. Each color cluster represents a different densest waterway.

The optimized trajectory set is generated by the DBSCAN trajectory clustering, and trajectories are roughly classified into incoming waterway, crossing waterway, and north–south routes. This allows us to extract the area of each trajectory to be predicted, and then each classified waterway is used as a dataset separately for subsequent data processing, as shown on Figure 6.

**Figure 7:**
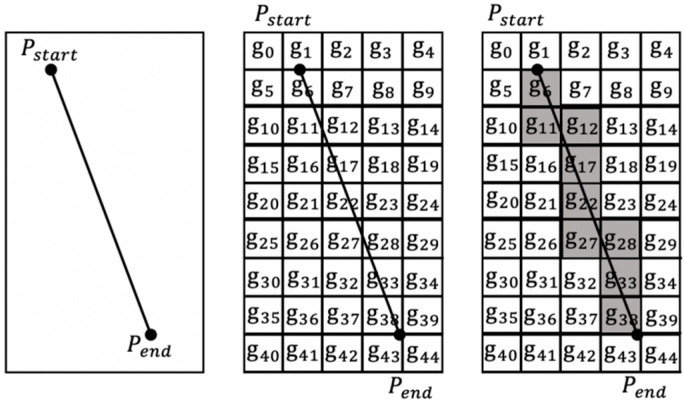
After gridding, the shaded area represents the grid for counting the number of times the ship has passed through the cross section.

**Figure 8:**
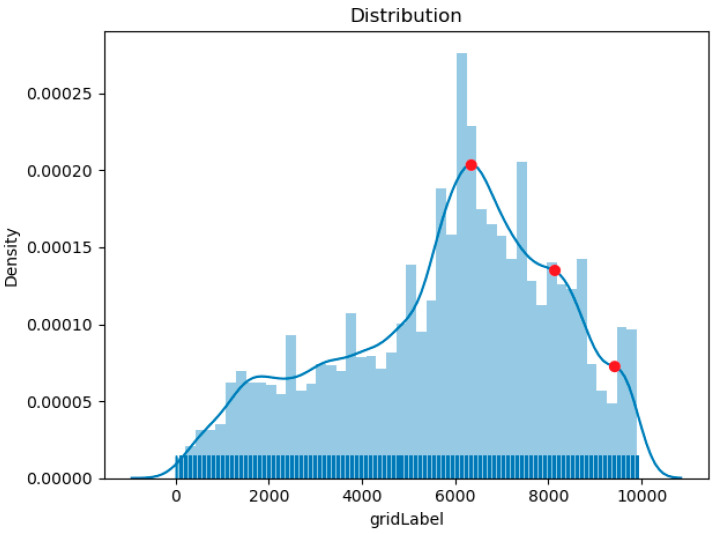
The gridded area shows the number of crossings recorded by each grid in the form of frequency.

This section uses statistical inferences on the crossing points of multiple ship trajectories through the cross section and extracts the trajectory with the characteristics of the trajectory cluster as the similarity measurement trajectory as shown in Figure 7. The specific process is as follows:

Step 1: Select a segment of clustered trajectory cluster data, classify the AIS trajectory point set according to MMSI, and connect the AIS trajectory points into a polyline with speed and direction.
Step 2: Select two coordinate points on the ship trajectory cluster to form a line as the cross section, and grid the surrounding position information into $g_{0}$, $g_{1}$,⋯, $g_{n}$, to record the crossing position of the ship trajectory.
Step 3: Use the line and line intersection algorithm to determine whether the ship’s trajectory passes through the cross section, count the number of crossing points the ship passes through the cross section, and store it in the grid id.
Step 4: The frequency at which the ship traverses the position is expressed in the form of probability density. The estimated value of the grid unit uses the Gaussian kernel function. The grid of the longitude and latitude coordinates corresponding to the peak of the probability density curve is selected as the Get used as crossing areas as shown in Figure 8.
Step 5: Intercept multiple times on the track clusters of the ship trajectory in this segment, repeat Step 4 to get the grid id that the ship is accustomed to traverse, until a trajectory is obtained that fully displays the features of the track in this segment.
Step 6: Use the most representative trajectory obtained in Step 5 as the similarity measurement trajectory. The specific similarity measurement method is introduced in Section 3.3.

### 3.3. Trajectory Similarity Measurement

**Figure 9:**
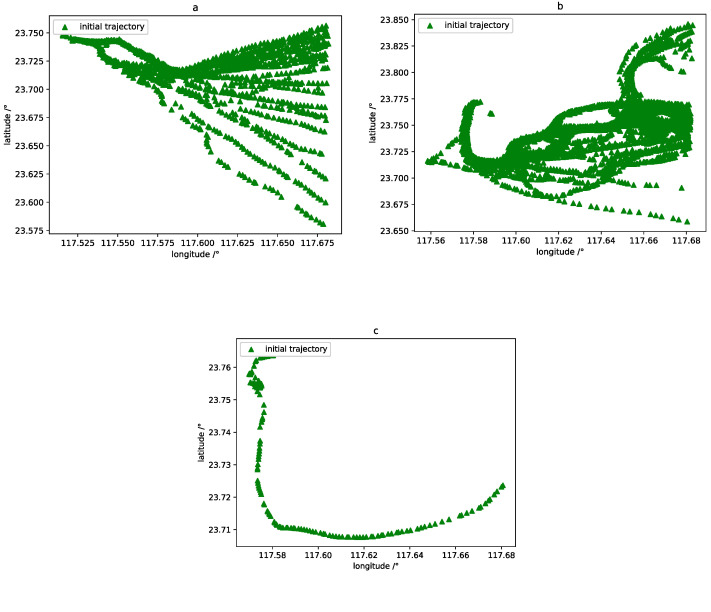
The three representative common vessel trajectories: (**a**) straight trajectory entering a port; (**b**) circuitous trajectory in narrow seas; and (**c**) trajectory with large turns in wide seas.

The quality of the incoming data has an important impact on vessel trajectory predictions. Due to the large difference in the shape of each trajectory, as shown in Figure 9, there are many redundant data that are not related to the target trajectory. To solve this problem, there is a need to obtain more reliable and useful datasets. A data preprocessing algorithm based on the Symmetrized Segment-Path Distance (SSPD) is applied [24]: First, let the historical trajectory after data preprocessing and the target trajectory be evaluated by calculating the similarity coefficient between each trajectory. Second, a filtering process is triggered according to the similarity coefficient. Finally, a subset of relevant trajectory data that meet the conditions is obtained. The SSPD model is described in detail below.

Most vessel trajectories are curves or straight, and trajectory similarity measurements mainly depend on the following conditions:

Nearly similarity shape
Physically closer to each other
Similar as a whole, rather than just similar subparts

**Figure 10:**
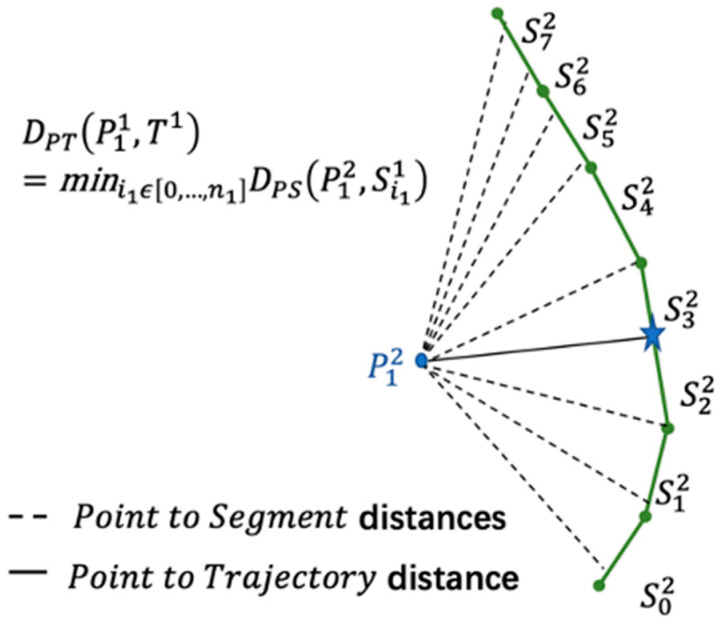
Distance from point $p_{1}^{2}$ to $T^{1}$. $S_{i}^{2}$ represents segments. The distance of $P_{1}^{2}$ between $S_{3}^{2}$ is the minimum.

**Figure 11:**
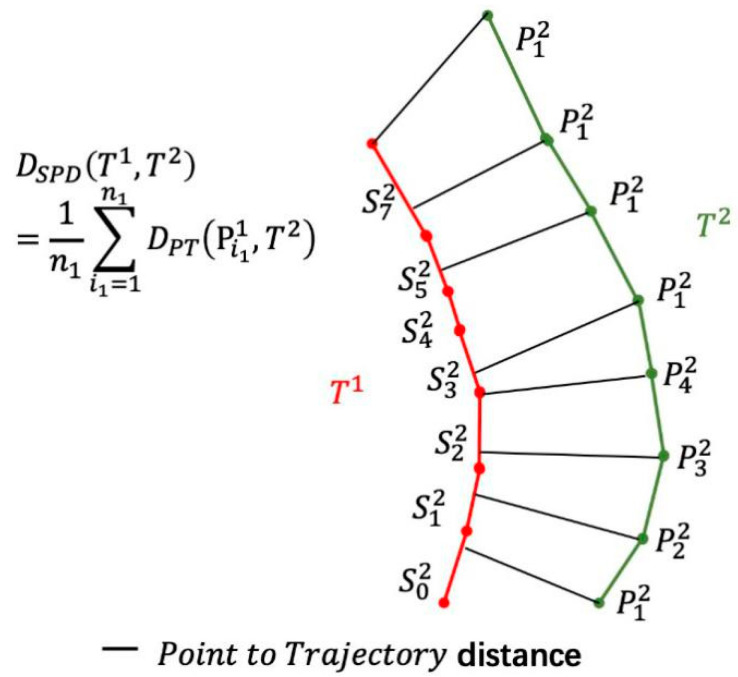
SPD distance from the trajectory $T^{1}$ to the trajectory $T^{2}$.

The definition of the SSPD is based on a point-to-segment distance, which is derived by the Hausdorff distance, $D_{pt}$, as shown in Figure 10. It is given by the minimum of distances between the points of all segments. The segmented-path distance from the trajectory $T^{1}$ to the trajectory $T^{2}$ is given by the average of all distances from each point that compose $T^{1}$ to the trajectory $T^{2}$, as shown in Figure 11.

Suppose one of the targets to be measured is $T_{s}{}^{1}$, and the other known trajectory is $T_{s}{}^{2}$. Since the SSPD algorithm is proposed based on the Hausdorff distance, according to the definition of the midpoint of the Hausdorff distance to the line segment, the distance between the line segment on the trajectory $T_{s}{}^{2}$ and the trajectory $T_{s}{}^{1}$ is given as

(11) $$D_{ps}\left(p_{i_{1}}^{1},s_{i_{2}}^{2}\right)=\{\begin{array}{c} \;\|p_{i_{1}}^{2}p_{i_{2}}^{2proj}\|{}_{2}\;,\;\;\;\mathrm{if}\;\;\;p_{i_{2}}^{2proj}\epsilon \;s_{i_{1}}^{1}\\ \min\left(\|p_{i_{2}}^{2}p_{i_{1}}^{1}\|{}_{2},\|p_{i_{2}}^{2}p_{i_{1}+1}^{1}\|{}_{2}\right),\;\;\;\mathrm{otherwise} \end{array}$$

where $p_{i_{2}}^{iproj}$ represents the orthogonal projection of the point $p_{k}^{i}$ on the trajectory segment $s_{l}^{j}$ and $\|p_{i_{2}}^{2}p_{i_{1}}^{1}\|{}_{2}$ represents the Euclidean distance between $p_{i_{2}}^{2}$ and $p_{i_{1}}^{1}$.

The distance from the point on the trajectory $T_{s}{}^{2}$ to the trajectory $T_{s}{}^{1}$ is defined as

(12) $$D_{pt}\left(p_{i_{1}}^{1},T_{s}{}^{1}\right)=\min_{i_{1}\epsilon \left[0,\ldots ,n_{1}\right]}D_{ps}\left(p_{i_{2}}^{2},s_{i_{1}}^{1}\right)$$

The segmented-path distance from the trajectory $T_{s}{}^{2}$ to the trajectory $T_{s}{}^{1}$ is defined as

(13) $$D_{SPD}\left(T^{1},T^{2}\right)=\frac{1}{n_{1}}\overset{n_{1}}{\underset{i_{1}=1}{\sum }}D_{pt}\left(p_{i_{1}}^{1},T^{2}\right)$$

The symmetric segmented-path distance from the trajectory $T_{s}{}^{2}$ to the trajectory $T_{s}{}^{1}$ is based on the segmented-path distance from the trajectory $T_{s}{}^{2}$ to the trajectory $T_{s}{}^{1}$ which can be written as

(14) $$D_{SSPD}\left(T^{1},T^{2}\right)=\frac{D_{SPD}\left(T^{1},T^{2}\right)+D_{SPD}\left(T^{2},T^{1}\right)}{2}$$

The smaller is the value of $D_{SSPD}\left(T^{1},T^{2}\right)$, the higher is the degree of similarity between $T_{s}{}^{1}$ and $\;T_{s}{}^{2}$.

Let the trajectory made of the grid processed as in Section 3.2 be target trajectory and compare the similarity with these trajectories ${\left[T_{s_{1}}\;T_{s_{2}}\;\cdots \;T_{n}\right]}^{T}$. The data preprocessing flow based on SSPD is shown in Algorithm 2. The similarity value of each known trajectory and the target trajectory is calculated by the SSPD algorithm; the similarity threshold is set according to the actual situation; and, among all similarity coefficients, the trajectory within the threshold is selected and used as the dataset.
**Algorithm 2:** Trajectory Similarity MeasurementInput: Trajectory dataset $Tr_{i}$ preprocessed by Algorithm 1, similarity threshold ε. Output: Trajectories $T_{sr_{i}}.$
1: for $T_{s_{i}}$ in ${\left[T_{s_{1}}\;T_{s_{2}}\;\cdots \;T_{n}\right]}^{T}$
2: calculate the distance $D_{SSPD_{i}}\left(T_{s_{i}},T_{s}\right)$ between $T_{s_{i}}$ and $T_{s}$
3: end for4: for $D_{SSPD_{i}}\left(T_{s_{i}},T_{s}\right)$ in $\left[D_{SSPD_{1}}\;D_{SSPD_{2}}\;\cdots \;D_{SSPD_{n}}\right]$ 5: save the trajectory which $D_{SSPD_{i}}$ < ε 6: end for
 Return: $\left[T_{sr_{1}}\;T_{sr_{2}}\;\cdots \;T_{sr_{m}}\right]$


## 4. GRU Model

**Figure 12:**
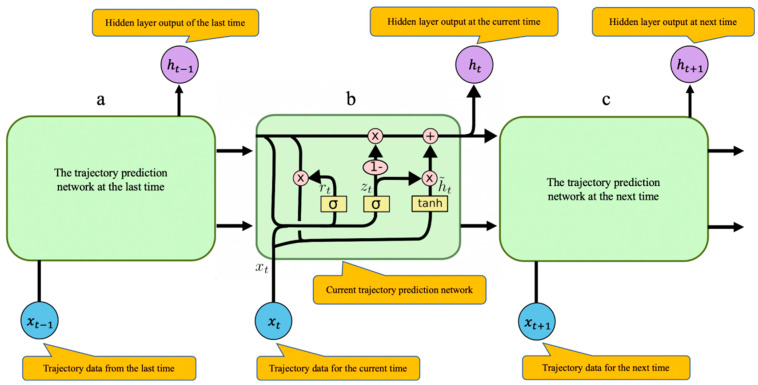
Schematic diagram of the trajectory prediction model based on the GRU neural network: (**a**) GRU unit at the last time; (**b**) GRU unit of the current time, which describes the detailed information of the transfer process; and (**c**) GRU unit at the next time.

A Recurrent Neural Network (RNN) is a type of recurrent neural network that takes sequence data as an input while cyclic units are connected in a chain [25] and that can deal with short-term prediction problems. However, it has been observed that RNN is prone to the problem of gradient disappearance when training the network. This leads us to introduce the GRU model initially introduced by Cho et al. [26] to improve the performance of an RNN network. The GRU structure contains the hidden layer state of the original RNN and is also a variant of LSTM. The GRU structure is similar to the LSTM model but computationally cheaper, as the GRU combines the forgotten gate and the input gate of the LSTM into a single update gate. The main advantage is that the GRU structure can store historical time-series information. It uses a gating mechanism to remember as long as possible previous trajectories, while simplifying the processing. It has a distinct hierarchical structure different from LSTM in the sense that GRU has fewer parameters than LSTM, but it can perform similarly to LSTM. The main principles behind the GRU structure is shown in Figure 12.

Let us introduce more formally the principles of the GRU neural network. For the hidden layer of the GRU neural network, given the input value $x_{t}\left(t=1,2,\cdots ,n\right)$, the value of the hidden layer at t is [27]:

(15) $$r_{t}=\sigma \left(W_{r}\cdot \left[h_{t-1},x_{t}\right]\right)$$

(16) $$z_{t}=\sigma \left(W_{z}\cdot \left[h_{t-1},x_{t}\right]\right)$$

(17) $${\tilde{h}}_{t}=\tanh\left(W_{\tilde{h}}\cdot \left[r_{t}*h_{t-1},x_{t}\right]\right)$$

(18) $$h_{t}=\left(1-z_{t}\right)*h_{t-1}+z_{t}*{\tilde{h}}_{t}$$

where σ is sigmoid $\left(x\right)=\frac{1}{1+e^{-x}}\;,\;\tanh\left(x\right)=\frac{e^{x}-e^{-x}}{e^{x}+e^{-x}},$ the calculation method [ ] indicates that two vectors are connected, ∗ means multiplying matrix elements, and · indicates matrix multiplications as follows:

(19) $$m*n=\left[\begin{array}{c} m_{1}\\ m_{2}\\ m_{3}\\ \vdots \\ m_{n} \end{array}\right]*\left[\begin{array}{c} n_{1}\\ n_{2}\\ n_{3}\\ \vdots \\ n_{n} \end{array}\right]=\left[\begin{array}{c} m_{1}n_{1}\\ m_{2}n_{2}\\ m_{3}n_{3}\\ \vdots \\ m_{n}n_{n} \end{array}\right]$$

Equations (9)–(12) show that the weight matrices at time t that the GRU neural network needs to train are $W_{z}$, $W_{r}$, and $W_{\tilde{h}}$, which are, respectively, composed of two weight matrices as follows:

(20) $$W_{z}=W_{zx}+W_{z\tilde{h}}$$

(21) $$W_{r}=W_{rx}+W_{r\tilde{h}}$$

(22) $$W_{\tilde{h}}=W_{\tilde{h}x}+W_{\tilde{h}\tilde{h}}$$

where $W_{zx}$, $W_{rx}$, and $W_{\tilde{h}x}$ are the weight matrix from the input value to the update gate, the reset gate, and the candidate value, respectively. $W_{z\tilde{h}}$, $W_{r\tilde{h}}$, and $W_{\tilde{h}\tilde{h}}$ are the weight matrix from the last candidate value to the update gate $z_{t}$, the reset gate $r_{t}$, and the candidate value $\tilde{h}$ respectively.

**Figure 13:**
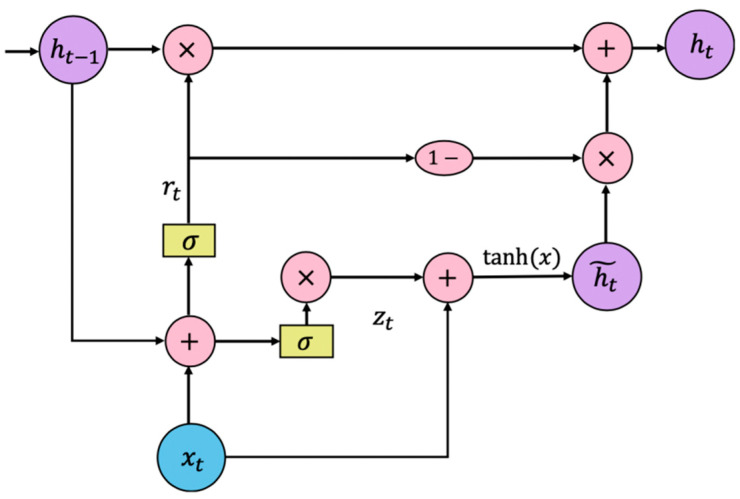
Schematic diagram of the GRU neural network hidden layer structure.

“1-” indicates that each element in the vector will be subtracted from 1. For the value of $z_{t}$ at time t, the larger is the value, the less the hidden layer value $h_{t}$ at time t−1 is affected by the hidden layer value $h_{t}$ at time t−1, and the more affected it is by the candidate value ${\tilde{h}}_{t}$ at time t. If the value of $z_{t}$ is close to 1, it means that the value $h_{t-1}$ of the hidden layer at t−1 does not contribute to the value $h_{t}$ of the hidden layer at time t. $z_{t}$ can better reflect the impact of the data with longer time intervals to the current moment in the time series sequence. The larger is the value $r_{t}$ at time t, the greater is the influence of the candidate value ${\tilde{h}}_{t}$ at time t on the hidden layer value $h_{t-1}$ at time t−1. If the value of $r_{t}$ is approximately null, it means that the hidden layer value $h_{t-1}$ at time t−1 does not contribute to the candidate value ${\tilde{h}}_{t}$ at time t, and $r_{t}$ can better reflect the impact of the data with short time intervals to the current time in the time series sequence. The GRU neural network hidden layer structure is shown in Figure 13.

GRU merges the forgotten gate layer $f_{t}$ and input gate $i_{t}$ of the LSTM unit into the $z_{t}$ of the GRU unit, as well as merges the hidden state and unit state of the LSTM network, together with the $r_{t}$, which is used to subject the degree of ignoring the status information of the previous time to control the flow of ship trajectory information. The updated gate is used to control how much status information of the previous moment is brought into the current state. Specifically, it combines the newly input trajectory information of the next time and the previous memory to determine what information is used in the current state to calculate the state information of the next time. The combination of GRU threshold structures retains the most important data to ensure that some information is not lost during long-term propagation. Due to the relatively simple structure of the GRU model, fewer parameters need to be trained, and it has the advantage of fast training speed during the training process.

## 5. Experiments and Analysis

The vessel trajectory datasets are classified after DBSCAN clustering, using the clusters of the north–south routes as the experimental dataset, which is divided into three parts: the training set, validation set, and test set. The training set is used to train the model. In the training process, the validation set is used to verify the performance of the model to improve its generalization ability [28]. The test set is used to generate some prediction results according to the input and output ways of N to 1 where the status vectors of the first N steps are taken as the input sequence and the status of the next time of the vessel as the output vectors. Then, the state of the first N minutes of a vessel is the original trajectory and the state of the vessel after N minutes is the one to be predicted. To ensure dimensionless interference between the data, the original time series is converted into the input sequence of the recurrent neural network after being normalized by

(23) $$X_{std}=\frac{x-x_{min}}{x_{max}-x_{min}}$$

where $X_{std}$ is the normalized data and $x_{min}$ is the minimum value of the sample. After normalization, the data are mapped in the interval [0, 1].

The result of output processed by the neural network in the interval [0,1] is denormalized and mapped to the original dimension level of the sample by

(24) $$X_{scaler}=x_{std}\left(x_{max}-x_{min}\right)+x_{min}$$

where $X_{scaler}$ is the data after denormalization.

To evaluate the results of GRU in vessel traffic flow prediction, LSTM is constructed as comparative experiments, and Mean Square Error (MSE) is selected as the model error analysis index during the training process as followed.

(25) $$MSE=\frac{1}{n}\overset{n}{\underset{i=1}{\sum }}{\left(y_{i}-{\hat{y}}_{i}\right)}^{2}$$

where $y_{i}$ is the ground-truth trajectory value and; ${\hat{y}}_{i}$ is the predicted value.

The selection of parameters is crucial for recurrent neural networks. Generally speaking, the more hidden layers are included in the GRU recurrent neural network, the stronger is the model’s performance and learning ability. The Adam optimizer [29], which is a stochastic gradient descent and adaptive learning rate optimization method, was used in the experiment. It can avoid the training process from falling into a local optimum situation. After determining the sampling rate as 1 in the experiment, the essential parameters in the network, batch size and the number of neurons, were experimentally compared and demonstrated to obtain the best parameter combination [30]. The selectable range of the batch size was {8, 16, 24, 32}, and the range of the number of neurons could be selected from {60, 80, 100, 120, 140, 160}.

The selection of parameters such as the neurons is shown in Table 2 and Table 3. As the number of batch size gradually increases, the calculation time gradually decreases. When the number of batch sizes is too small or large, the generated errors are excessive. When the quantity of neurons is 16 and 24, respectively, the calculation time is similar, and the former error is smaller, thus a valuable number of batch size is given as 16. As shown in Table 3, as the number of neurons gradually increases, the consumption time also gradually increases. When the number of neurons increases from 60 to 120, the prediction error gradually decreases. When the number of neurons increases further, the error increases, thus, for the neurons, it is more reasonable to set the number at 100.

**Figure 14:**
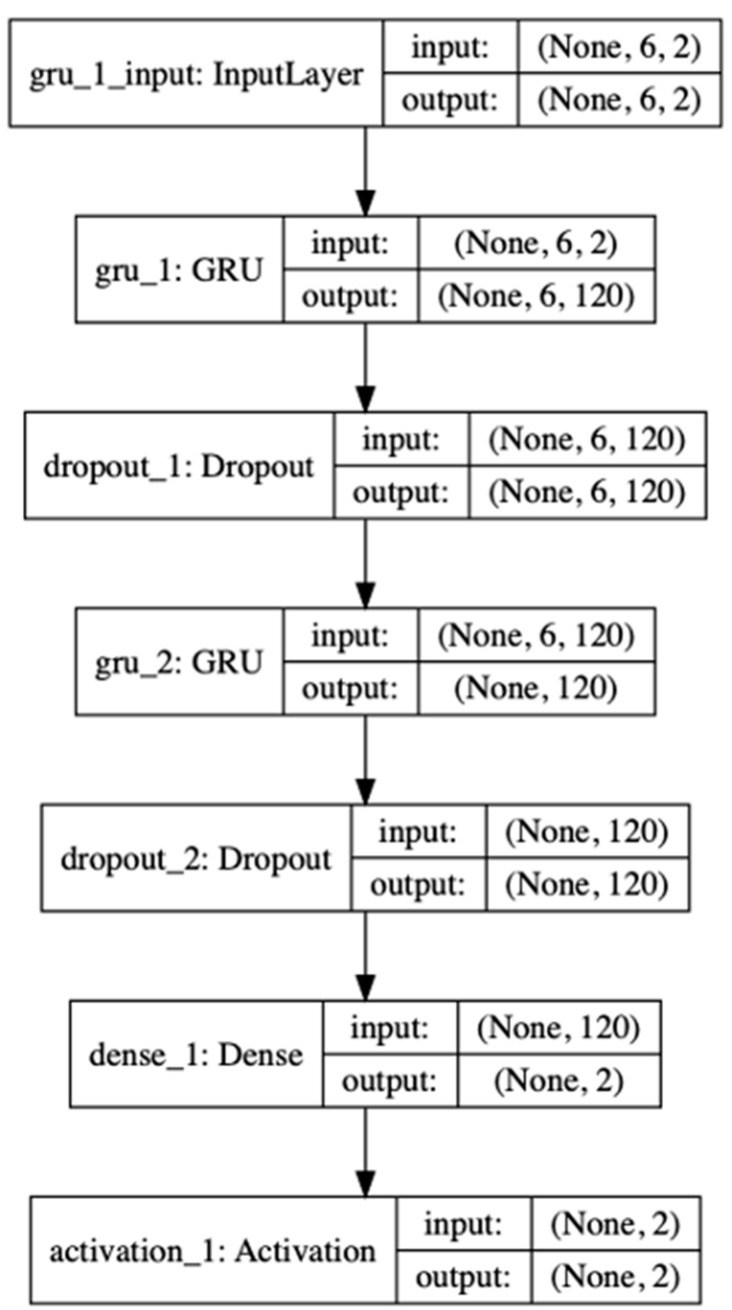
Plot of all parameters setting of GRU recurrent neural network model.

After several experimental tests and parameter comparisons, the GRU construct consists of the following parts: fully connected layer, an activation layer, two GRU layers, and a method of drop out to construct the GRU recurrent neural network. The second layer gru_1 and fourth layer gru_2 are the GRU layers, each containing 120 hidden units; the third and fifth layers are the drop out method, which can not only ensure that the model maintains the robustness of the model when information is lost during training, but it can also be used for regularization, reducing the weight connection, increasing the robustness of the network model in the event of missing individual connection information; and the fifth layer is a fully connected layer, which contains two neurons. The visualization of the GRU neural network is shown in Figure 14.

In the training process, as shown in Figure 14, both models iterate quickly, and the training loss value can quickly obtain a better convergence effect. When the number of training rounds is about 30, GRU has reached the extremum; LSTM as a control test reached the extremum value at 50 rounds. The improved GRU model based on LSTM can converge to the extreme point more quickly than the LSTM model in the training phase. The fast convergence speed is mainly due to the simplified GRU gate structure for the gate design of the LSTM structure.

**Figure 15:**
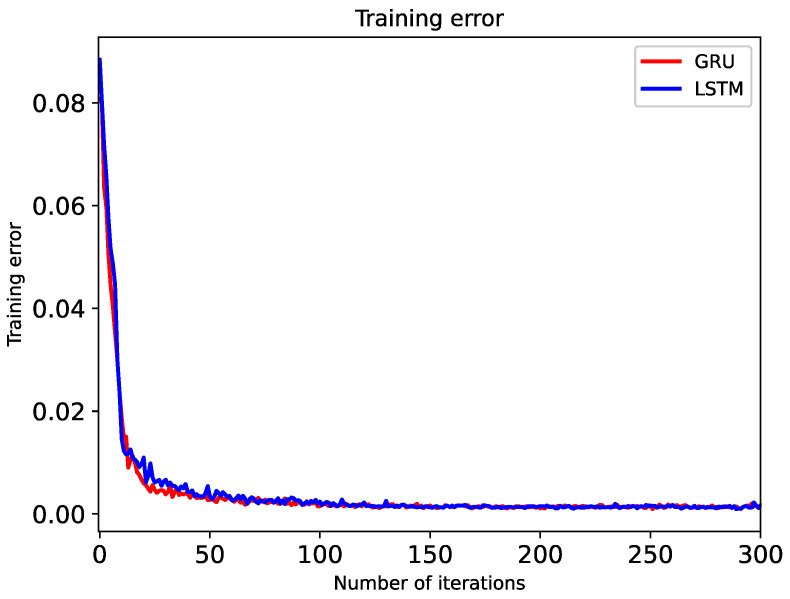
Plot of comparison of iterative convergence of two models.

**Figure 16:**
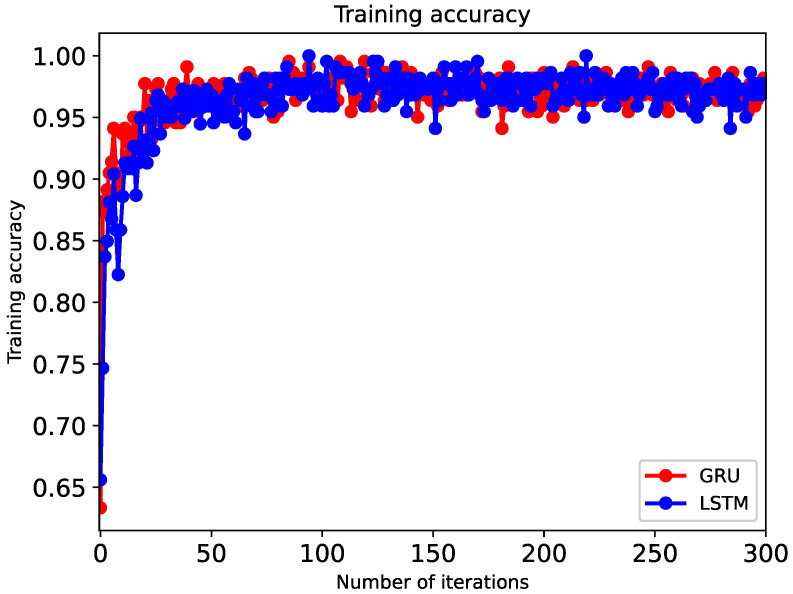
Plot of comparison of prediction accuracy diagram of two models.

**Figure 17:**
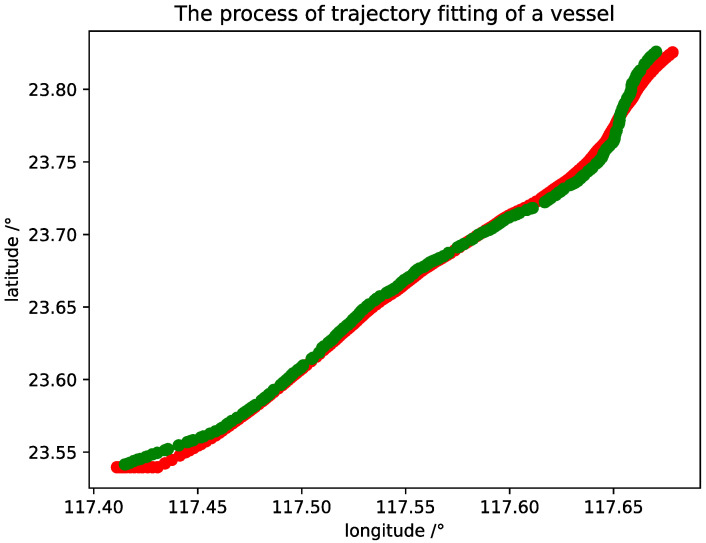
Plot of comparison of a real trajectory and fitting trajectory uses the GRU model.

The model training is shown in Figure 15 and Figure 16, and one of the results of the trajectory fitting process is presented in Figure 17, from which we can see that the fitting effect is satisfactory. When the training phase reaches about 30 rounds, the accuracy of the GRU model reaches 96%; the accuracy rate from the 200th round to the 300th round reaches about 98%; and then it gradually smooths, which shows that the efficiency of GRU is better than that of LSTM.

**Figure 18:**
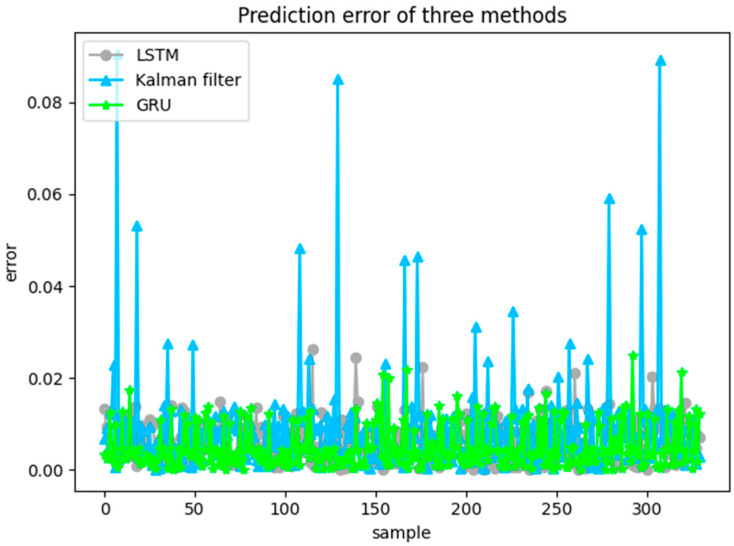
Prediction errors of the three methods calculated by equation (25): gray solid circle markers indicate the prediction results of LSTM, blue solid triangle indicate those of EKF, and the green solid stars markers denote those of GRU model.

A total of 330 sets of trajectory data was used to predicted using LSTM, Extended Kalman Filter (EKF), and GRU models as shown in Figure 18. From the experimental results, the deep learning method is better than the traditional statistical-based method EKF; EKF’s prediction results are good, but, compared with deep learning, the prediction error is not stable and much bigger errors appear due to the strong nonlinearity of the data. Based on the same framework, the prediction results of GRU and LSTM are relatively similar, and the errors are basically stable within 0.02. With similar prediction accuracy, it can be seen in Table 2 and Table 3 that the calculation efficiency of GRU model is slightly better than LSTM.

**Figure 19:**
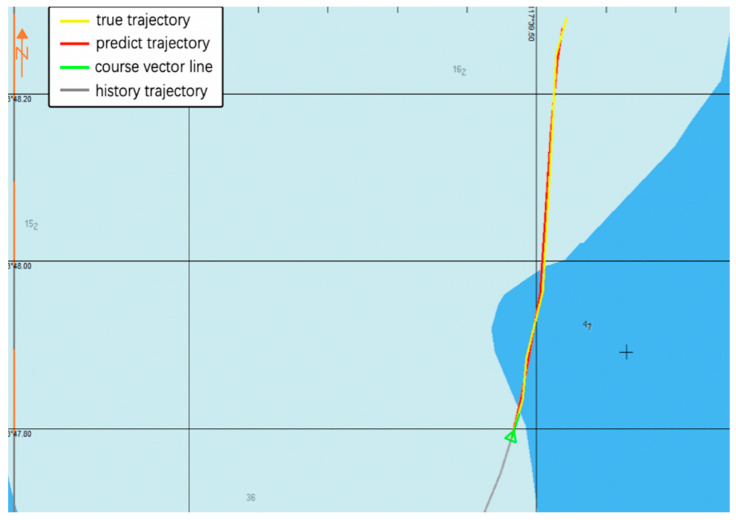
Visualization of the vessel’s real trajectory and predicted trajectory that uses the GRU model.

**Figure 20:**
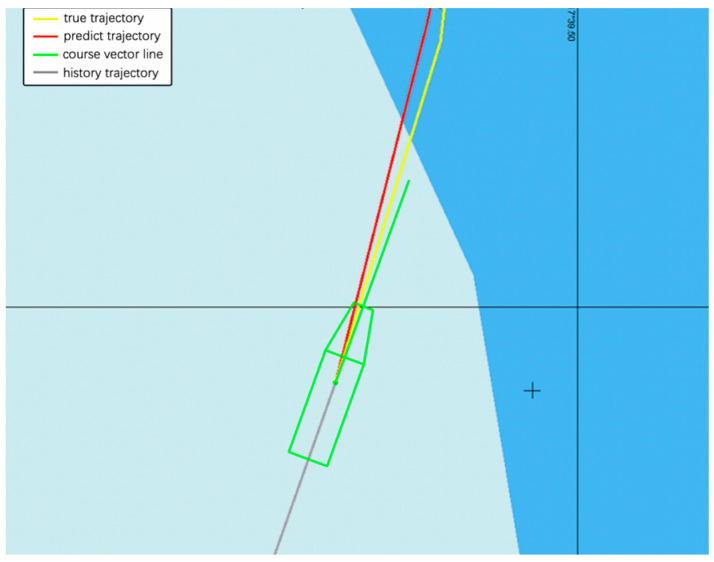
A detailed representation of the vessel’s real trajectory and predicted trajectory that uses the GRU model.

A vessel trajectory is selected from the historical AIS data of Zhangzhou Port, China, visualized in the Electronic Chart Display and Information System (ECDIS), as shown in Figure 19 and Figure 20. The prediction trajectory of the GRU model is illustrated, and prediction results are visualized. The gray line represents the observed history trajectory until the current time, the red line denotes the predicted trajectories, the yellow line represents the ground-truth trajectory at the 30th minute of the path, while the green line represents the course vector bar of the vessel. There is a turning point in the trajectory, which can verify that the predictive performance of our model can be applied to nonlinear data. The predicted trajectory almost overlaps with the real trajectory, and a good prediction effect is achieved.

## 6. Conclusions

This paper introduces a ship trajectory prediction framework based on a recurrent neural network. One of the peculiarities of our approach is to take into account vessel navigation errors due to receiving and sending data processes. The vessel trajectory prediction model is divided into data preprocessing, clustering analysis, similarity measurement, and model analysis. The objective of the first three modules is to extract high-quality datasets. The model was tested with a series of real data and different parameter combinations within a reasonable range o train the neural network and predict vessel trajectories is the most valuable way. A comparative experiment was conducted with LSTM that is considered as a valuable solution. The experiments showed that the vessel trajectory prediction model based on the GRU model has good prediction accuracy similar to that of LSTM, but it has the advantage of being computationally more efficient. It is more suitable for the requirements of immediate and early warnings of maritime navigation and can provide appropriate decisions for intelligent vessel navigation systems and VTS. LSTM is similar to GRU, and it is less effective in long-distance trajectory prediction. On the basis of improving the calculation efficiency, if the accuracy of long-distance trajectory prediction can be improved, it will be of great help to the early warning of ship navigation hazards. While current trajectory prediction model is based on a recursive call model, long-distance vessel trajectories are still computationally expensive (e.g., predicting the vessel’s arrival at the port), which will be considered in further work.

## Figures and Tables

**Table 1:** Comparison before and after data processing.

Data	Total Coordinates (Points)	Number of Ships	Number of Voyages
Raw Data	7,577,484	13,437	1,004,137
After Data Preprocessing	2,134,324	7231	78,531

**Table 2:** Comparison of the experimental influence of different parameters of batch size on the two models.

Model	Metrics	Number of Batch Size			
		8	16	24	32
GRU	MSE	1.38 × 10^−^^3^	1.03 × 10^−^^3^	1.47 × 10^−^^3^	2.87 × 10^−^^3^
	Time Resuming/s	205.23	124.03	108.00	87.14
LSTM	MSE	1.26 × 10^−^^3^	1.03 × 10^−^^3^	1.46 × 10^−^^3^	3.03 × 10^−^^3^
	Time Resuming/s	221.24	128.32	103.21	90.34

**Table 3:** Comparison of the experimental influence of different parameters of neurons on the two models.

Model	Metrics	Number of Neurons					
		60	80	100	120	140	160
GRU	MSE	6.72 × 10^−^^3^	3.01 × 10^−^^3^	1.93 × 10^−^^3^	1.02 × 10^−^^3^	4.59 × 10^−^^3^	4.63 × 10^−^^3^
	Time Resuming/s	86.99	103.43	124.03	135.97	154.53	176.74
LSTM	MSE	6.34 × 10^−^^3^	2.89 × 10^−^^3^	1.93 × 10^−^^3^	1.02 × 10^−^^3^	4.03 × 10^−^^3^	4.27 × 10^−^^3^
	Time Resuming/s	88.43	107.34	124.59	133.62	153.43	173.75
